# One-Dimensional Control System for a Linear Motor of a Two-Dimensional Nanopositioning Stage Using Commercial Control Hardware

**DOI:** 10.3390/mi9090421

**Published:** 2018-08-22

**Authors:** Lucía Candela Díaz Pérez, Marta Torralba Gracia, José Antonio Albajez García, José Antonio Yagüe Fabra

**Affiliations:** 1 I3A, University of Zaragoza, C/María de Luna 3, 50018 Zaragoza, Spain; jalbajez@unizar.es (J.A.A.G.); jyague@unizar.es (J.A.Y.F.); 2 Centro Universitario de la Defensa, Ctra. Huesca s/n, 50090 Zaragoza, Spain; martatg@unizar.es

**Keywords:** positioning platform, Halbach linear motor, commercial control hardware

## Abstract

A two-dimensional (2D) nanopositioning platform stage (NanoPla) is in development at the University of Zaragoza. To provide a long travel range, the actuators of the NanoPla are four Halbach linear motors. These motors present many advantages in precision engineering, and they are custom made for this application. In this work, a one-dimensional (1D) control strategy for positioning a Halbach linear motor has been developed, implemented, and experimentally validated. The chosen control hardware is a commercial Digital Motor Control (DMC) Kit from Texas Instruments that has been designed to control the torque or the rotational speed of rotative motors. Using a commercial control hardware facilitates the applicability of the developed control system. Nevertheless, it constrains the design, which needs to be adapted to the hardware and optimized. Firstly, a dynamic characterization of the linear motor has been performed. By leveraging the dynamic properties of the motor, a sensorless controller is proposed. Then, a closed-loop control strategy is developed. Finally, this control strategy is implemented in the control hardware. It was verified that the control system achieves the working requirements of the NanoPla. It is able to work in a range of 50 mm and perform a minimum incremental motion of 1 μm.

## 1. Introduction

Positioning stages are becoming fundamental devices in nanotechnology and nanomanufacturing processes [1,2], where they act as a supplementary unit for measuring or manipulating samples [3,4]. Depending on the application, a certain combination of working range and metrological performance is required [5]. To obtain effective positioning, several metrological systems are currently available [6,7,8]. These systems have been designed for demanding and accurate operations. Nevertheless, their measuring and positioning range is often very limited [9,10]. Other applications such as measuring or manipulating solar cells or silicon wafers require working with larger areas in a planar part, where cutting of specific samples may be necessary. Therefore, the nanotechnology industry is demanding not only more accurate positioning systems but also larger working ranges [11]. Within this line of research, a nanopositioning platform stage (NanoPla) has been developed and manufactured at the University of Zaragoza [12,13]. It is expected to provide effective positioning at the nanometre scale inside a large working range of 50 mm × 50 mm. Its first application integrates an atomic force microscope (AFM) as a suitable technique for micro- and nanometrology [14], due to the high vertical as well as lateral resolution in the topographic characterization task of specimens.

Depending on their structure, nanopositioning stages can be classified into stages with stacked linear axes and plane stages. Stages with stacked linear axes are characterized for long kinematic chains with an unfavourable force transfer behaviour [15,16]. Whereas the absence of linear motion in plane stages minimizes geometrical errors and presents many other advantages in precision engineering [17]. For these reasons, it has already been implemented in multiple systems [18,19].

In the NanoPla design, the principles of precision engineering have been applied, including planar motion. However, planar motion conditions the actuator selection, since the motor design or its guiding system should not impede the displacement of the motor along the orthogonal direction of its driving axes. Halbach linear motors [20] suppose a solution to this issue, whose movement in the 2D plane is only limited by the size of its winding area. Other advantages of Halbach linear motors are that they provide non-contact motion and, in addition to the propulsion force, they generate a levitation force. Although one of the design criterions of the NanoPla is to implement as many commercial devices as possible, unguided Halbach linear motors are not commercialised yet. Therefore, they have been custom-made for this application due to the advantages of performing accurate and long travel range positioning.

The fact that the use of this kind of Halbach linear motors is not yet widespread means that there is no available commercial solution for the driving task. In other positioning stages described in the literature [6,21], the control hardware and software were specifically designed and built for this purpose. Nevertheless, as was mentioned, one of the targets of the NanoPla design is to develop it with commercial devices when possible, which will facilitate a future industrial applicability of the developed system. Thus, a commercial generic solution for the hardware has been chosen: a Digital Motor Control (DMC) Kit from Texas Instruments (Dallas, TX, USA). This control hardware has been designed for rotary permanent magnet synchronous motors (PMSM), where the aim is to control the rotation speed or the torque generated. According to the literature, the integration of completely generic control hardware with linear motor actuators is a novelty that is presented in this study. Such integration presents many limitations that need to be overcome by optimizing the control system design. Nevertheless, this has been done in this work always by using the available options of the control hardware modules. The hardware has not been modified and no additional electronic has been required. The use of only one commercial hardware and no custom-made electronics facilitates the applicability and replication of the developed control strategy, which is in line with the targets of the NanoPla design. This work can be very useful for other developers willing to implement commercial devices for the control of linear motors.

This article presents and experimentally validates a challenging one-dimensional (1D) control system for a custom-made Halbach linear motor that works as an actuator in the two-dimensional (2D) long working range NanoPla. The control system is characterized by the integration of a commercial solution hardware which is commonly implemented with rotary actuators. Thus, this paper first presents an overview of the NanoPla, which is necessary to define the working requirements of the control system. Secondly, the working principle of Halbach linear motors is described, and the materials used in this work are presented. Then, a dynamic characterization of these motors is performed, and a sensorless open-loop solution is proposed. Afterwards, the 1D control strategy is defined, and the proposed control strategy is implemented in the chosen commercial control hardware. Finally, the experimental results are shown and conclusions are withdrawn.

## 2. Two-Dimensional Nanopositioning Platform Stage (NanoPla) Overview

**Figure 1:**
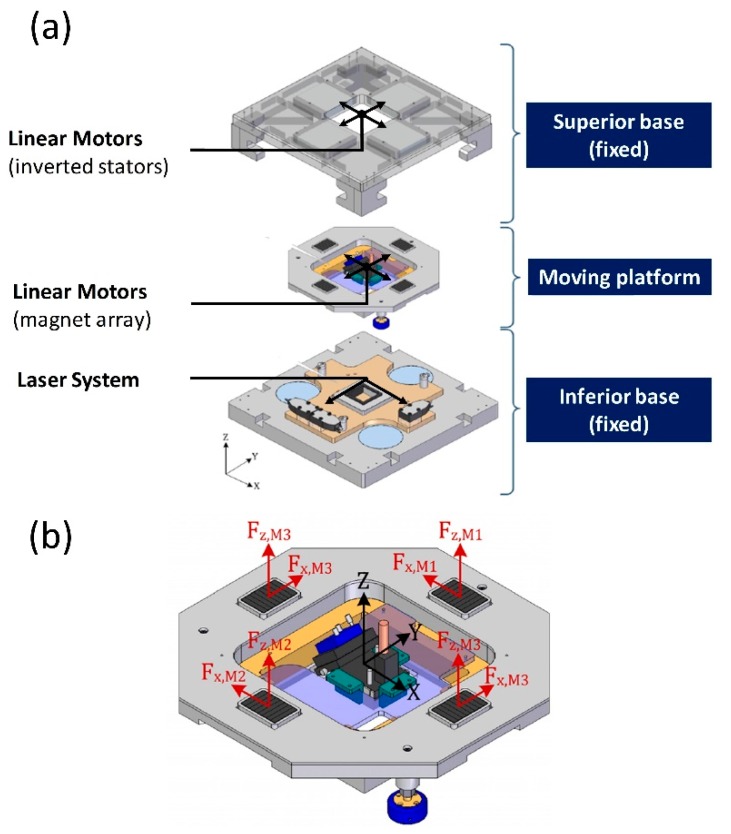
(**a**) Nanopositioning platform (NanoPla) prototype; (**b**) vertical and horizontal forces generated by the motors in the moving platform.

As shown in Figure 1a, the NanoPla consists of a three-layered architecture: an inferior and a superior base that are fixed, and a moving platform that is placed in the middle. Three air bearings lift the moving platform and levitate it. The planar motion is performed by four Halbach linear motors that are symmetrically assembled in an inverted position. In other words, the stators are fixed to the superior base, and the magnet arrays are assembled to the moving platform (see Figure 1a). The horizontal forces of each pair of parallel motors will move the platform in the X and Y direction, as Figure 1b shows. In addition, the vertical forces of the four motors will favour the levitation of the moving platform. A 2D laser interferometer system works as positioning sensor. The laser heads are fixed to the inferior base, and the mirrors are placed in the moving platform. In addition, in the NanoPla, a two-stage scheme has been applied. That means that the XY-long range positioning of the moving platform is complemented by an additional fine nanopositioning system for the more demanding scanning operations. This second stage is a commercial piezo-nanopositioning device with a working range of 100 × 100 × 10 μm^3^, which increases the number and variety of applications of the NanoPla.

The first device that is going to be integrated in the NanoPla is an AFM, which will be placed on the moving platform. The NanoPla will position the AFM along the working range of 50 mm × 50 mm. The sample will be placed in the commercial piezo-nanopositioning stage fixed to the inferior base. During the scanning task, the moving platform and the AFM will be static (air bearings off) and the commercial nanopositioning stage will perform the fine motion of the sample.

A preliminary modelling of the 2D positioning control of the NanoPla was presented in [22] with a different approach. The input currents are controlled independently, which is not possible with the commercial control hardware solution proposed. Nevertheless, the control strategy requirements were initially defined. According to this, the 1D linear motion control strategy must be able to work in a range of 50 mm. In addition, the positioning error must be at least on order of magnitude smaller than the maximum XY range of the commercial piezo-nanopositioning stage, i.e., 10 µm. The settling time is not critical. Finally, other considerations are related to the transient behaviour of the positioning response. In addition, oscillation should be avoided.

## 3. Halbach Linear Motors

The linear motors used as actuators in the NanoPla were developed by Trumper et al. [20] and custom-made in the Center for Precision Metrology of the University of North Carolina at Charlotte (Charlotte, NC, USA). They consist of a Halbach permanent magnet array and three-phase ironless coils (stator). In this section, the motor law and the commutation law that define the working principle of the motor are described.

### 3.1. Motor Law

**Figure 2:**
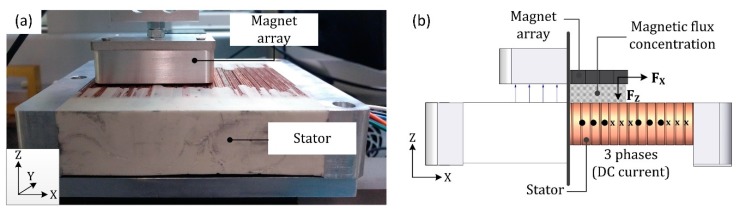
(**a**) Halbach motor (magnet array and stator); (**b**) graphical representation of the dual forces generated by the Halbach motor.

In a Halbach array of permanent magnets, the configuration of the magnets augments the magnetic field generated on one side and nullifies the magnetic field on the other side. That is, the rotating pattern of the permanent magnets forces the cancellation of magnetic components resulting in a one-sided flux. In a Halbach linear motor (Figure 2a), this flux is concentrated between the magnet array and the stator. When a DC current flows through the coils of the stator, these currents interact with the magnetic field of the magnet array. The electromagnetic interaction generates two orthogonal forces: one is horizontal (F_x_) and the other is vertical (F_z_), as can be seen in Figure 2b.

The direction and amplitude of the two forces depend on the relative position between the magnet array and the coils as well as on the magnitude of the DC phase currents flowing through the coils. The motor law (Equation (1)) represents the mathematical relationship between the generated forces (F_x_ and F_z_), the input phase currents (I_a_, I_b_ and I_c_) and the relative position between the stator and the magnet array (x_0_), along the axis of movement.

(1) $$\left[\begin{array}{c} F_{x}\;\\ F_{z} \end{array}\right]=A\left[\begin{array}{ccc} \cos\left(kx_{0}+\varphi \right) & \cos\left(kx_{0}-\frac{2\pi }{3}+\varphi \right) & \cos\left(kx_{0}+\frac{2\pi }{3}+\varphi \right)\\ \sin\left(kx_{0}+\varphi \right) & \sin\left(kx_{0}-\frac{2\pi }{3}+\varphi \right) & \sin\left(kx_{0}+\frac{2\pi }{3}+\varphi \right) \end{array}\right]\left[\begin{array}{c} I_{a}\\ I_{b}\\ I_{c} \end{array}\right]$$

In Equation (1), A and k are constant parameters of the motor. The constant of the motor, A, depends on design parameters of the motor, as represented in Equation (2). On the other hand, k is the fundamental wave number, which is calculated according to Equation (3), where l is the pitch or the spatial period of the array wavelength. In Table 1, a description of all these parameters is provided [23].

(2) $$A=N_{m}\eta_{0}\mu_{0}M_{0}Ge^{-kz_{o}\;}$$

(3) $$k=\frac{2\pi \;}{l}$$

Their values were calculated first theoretically and then experimentally in a previous work [24]. This was done by measuring with a load cell, along the travel range of the motor, the vertical and horizontal force generated by certain known phase currents. These results are shown in Table 2. The initial position x_0_ = 0 can be adjusted by changing the phase difference φ in Equation (1). φ must have the same value in the F_x_ row as in the F_z_ row because the two forces are orthogonal. In this paper, for simplicity reasons, the value of φ will be considered null. φ acts as an initial offset and, thus, this assumption only affects the absolute initial position of the motor but not the results.

### 3.2. Commutation Law

The commutation law is defined as the inverse of the motor law (Equation (1)), and it allows the calculation of the phase currents that are required in order to generate a certain F_x_ and F_z_ in a specific position. On the basis of Equation (1), the phase currents can be determined with one degree of freedom (three unknowns, two equations). As there are three input currents, one more equation needs to be considered to uniquely determine them. In [25], an additional constraint was proposed for power minimization, which is possible due to the fact that the control strategy acts independently on the input currents using linear transconductance power amplifiers built for that purpose. By contrast, this paper proposes the use of a generic DMC Kit from Texas Instruments. This control hardware imposes a star-connection on the phases of the motor, which adds an additional constraint (Equation (4)) that prevents the control of the three currents independently. Thus, it also impedes the implementation of the power minimization constraint.

(4) $$I_{a}+I_{b}+I_{c}=0$$

Therefore, combining Equations (1) and (4) and considering φ = 0, the commutation law for the case of this study is defined as in Equation (5):

(5) $$\left[\begin{array}{c} I_{a}\\ I_{b}\\ I_{c} \end{array}\right]=\frac{2}{3A}\left(\begin{array}{cc} \cos kx_{0} & \sin kx_{0}\\ \cos\left(kx_{0}-\frac{2\pi }{3}\right) & \sin\left(kx_{0}-\frac{2\pi }{3}\right)\\ \cos\left(kx_{0}+\frac{2\pi }{3}\right) & \sin\left(kx_{0}+\frac{2\pi }{3}\right) \end{array}\right)\left[\begin{array}{c} F_{x}\\ F_{z} \end{array}\right]$$

## 4. Experimental Setup and Hardware Description

**Figure 3:**
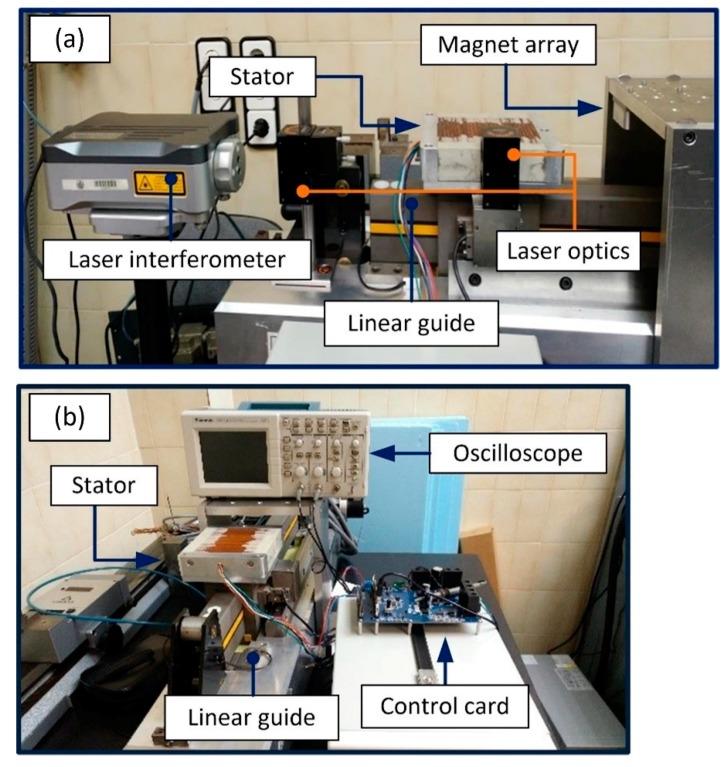
Lateral (**a**) and front (**b**) view of the experimental setup for the implementation of the control system of one linear motor.

Before the implementation of the control system designed in this work into the two-dimensional NanoPla, its validation is firstly performed in a separate experimental setup. In this manner, the experimental validation has been carried out in a metrology laboratory with standard conditions of temperature 20 ± 1 °C and humidity 50–70% controlled 24/7. The scheme of the experimental setup is shown in Figure 3. This setup installs one of the linear motors of the NanoPla and the same DMC Kit that will be implemented in the NanoPla. A pneumatic 1D-linear stage was used to imitate the frictionless motion of the NanoPla. The stator of the linear motor is mounted over the pneumatic linear guide, and the magnet array is fixed to the bridge part. The actuator is connected to the three-phase power stage of the control hardware, while the control card is connected to a computer by a USB port. As positioning sensor, a laser interferometer system has been used (i.e., laser head source and reflectors). The laser system is also connected to the computer. In addition, an oscilloscope has been used to monitor the signals of the control hardware.

As can be observed in Figure 3, in this preliminary setup, the stator is the moving part while the magnet array is static, in contrast to the design of the NanoPla. Nevertheless, the relative motion between parts is the same in both cases. Therefore, this does not affect the design of the control system nor the experimental validation.

As stated, this work proposes to facilitate the control issue by implementing a commercial solution for the control hardware. The selected device to perform the control is a DMC Kit (DRV8302-HC-C2-KIT) from Texas Instruments. This control hardware is designed to operate with generic rotary permanent magnet synchronous motors. It provides closed-loop digital control feedback, analogue integration and comprises a microcontroller unit (MCU) and the inverter stage that generates the phase voltages. The MCU is a C2000 microcontroller and is able to perform real time control by working with 32-bit data. The control hardware is able to generate three phase voltages. That means that in the NanoPla each motor will need one control DMC Kit.

A Renishaw XL-80 laser interferometer (Renishaw, Gloucestershire, UK) has been integrated to provide the position feedback. The readouts of the laser system are sent to the computer and then from the computer to the control card by a serial communication interface. The Renishaw XL-80 laser system has a resolution of 1 nm, and the measured noise under laboratory controlled conditions has a range of 400 nm. The purpose of this work is to develop, integrate and validate a 1D control strategy for one linear motor so that this control system can be implemented later in the four linear motors of the NanoPla for a 2D movement. Renishaw XL-80 laser system performance is similar to the laser system of the NanoPla, and it is perfectly suitable for this validation.

In contrast, the NanoPla includes a 2D laser system that belongs to the Renishaw RLE10 laser interferometer family. It consists of a laser unit (RLU), two sensor heads (RLD), two plane mirrors (one per axis), and an environmental control unit (RCU). In addition, an external interpolator improves the resolution to 1.58 nm. The measured noise of this system is 20 nm. In [26] an analysis of the performance of the NanoPla 2D laser system was presented and its suitability as positioning sensor was confirmed. This laser system will be used once the 2D positioning system is implemented in the NanoPla.

## 5. Dynamic Characterization

Now the driving actuators and the experimental setup have been described, in this section a dynamic characterisation of the system is performed. In other work that introduces the use of Halbach linear motors for metrology applications [20], electromechanical modelling was presented. In contrast, this section focuses on observing and understanding the dynamic behaviour of the motor under the electromagnetic forces that are generated when a DC current flows through the coils. This dynamic characterisation allows the definition of an open-loop control system, which will facilitate the design of the closed-loop control strategy described in the next section. Firstly, the conditions of the equilibrium of the system are studied. After defining the equilibrium state, a sensorless controller is developed. This controller moves the motor by varying the force distribution along the axis, and it does not require a positioning feedback sensor.

### 5.1. Equilibrium Position

**Figure 4:**
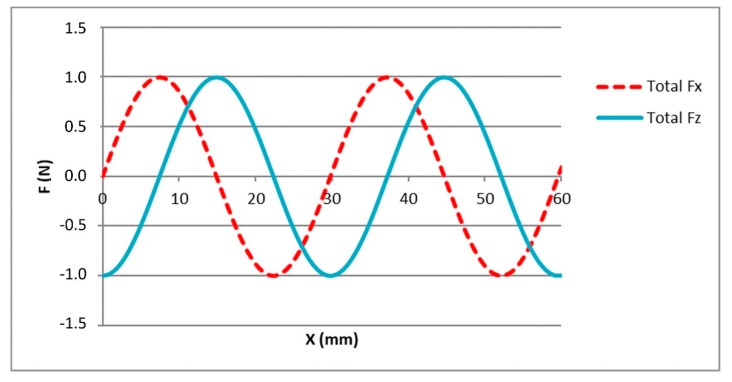
F_x_ and F_z_ along the axis of movement, for I_a_ = 0 A; I_b_ = 0.3593 A; I_c_ = −0.3593 A.

In the system under study, the only forces that act on the motor are the orthogonal electromagnetic forces F_x_ and F_z_, F_x_ being the only propulsion force that acts along the axis of movement. As mentioned in Section 3, when certain phase currents flow through the stator, F_x_ and F_z_ are generated, and their magnitude depends on the relative position between stator and magnet array. Figure 4 represents the sinusoidal shape of the horizontal force (F_x_) and the vertical force (F_z_) generated by certain phase current values along the axis of movement (x_s_).

The motor will remain motionless once it arrives at a position x_0_ where the propulsion force is null; that is, the equilibrium position. As can be observed in Figure 4, in each magnetic spatial period (pitch: l = 29.778 mm), there are two equilibrium positions. For instance, in the first pitch, F_x_ is equal to 0 N at the positions x_s_ = 0 mm and x_s_ = 14.889 mm. However, these two equilibrium positions have different characteristics. The second one, where the slope is negative, is a stable equilibrium position. As can be seen in Figure 4, if a perturbation displaces the motor from this stable equilibrium position, then the electromagnetic force pushes it forwards if the displacement is negative or backwards if the displacement is positive, always returning it to the stable equilibrium position. On the contrary, where the slope is positive, there is an unstable equilibrium position, where a small disturbance moves the motor away from its position to the nearest stable equilibrium position. According to Figure 4, at the stable equilibrium position, the value of the vertical force F_z_ is maximum and positive, while at the unstable equilibrium position, the value of F_z_ is minimum and negative.

In the NanoPla, once the motor arrives to the target or reference position (x_s_ = x_ref_), it must remain motionless (F_x_ = 0). In addition, the magnet arrays are fixed to the moving part that is levitating by means of three air bearings. In order to leverage the vertical force generated by the motor, F_z_, must be positive (Figure 2), favouring the levitation by lifting the magnet array. In other words, the target position must fulfil the conditions of a stable equilibrium position. For the experimental validation presented in this work, the target value of F_z_ has been defined as 1 N.

### 5.2. Electromagnetic Sensorless Controller

As stated in the previous subsection, according to the working conditions of the NanoPla, when the motor achieves the target position, it must be in a stable equilibrium state. By introducing the conditions of the stable equilibrium (F_x_ = 0 and F_z_ = 1 N) for a particular desired target position (x_s_ = x_ref_) in the commutation law (Equation (5)), the required phase currents that create this state can be calculated. When these currents flow through the coils, the electromagnetic forces are generated. Therefore, by combining the phase currents, the equilibrium state can be created at any desired position. Then, the horizontal force moves the motor to the stable equilibrium position (x_ref_) where it is maintained under small perturbations.

**Figure 5:**
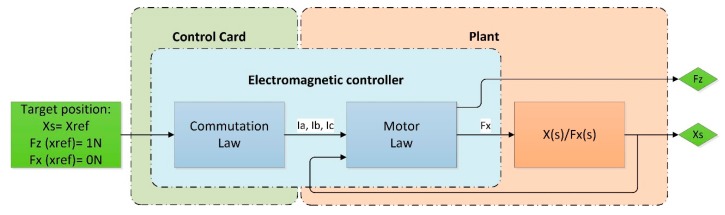
Scheme of the linear motor system in an open-loop system.

Thus, when the phase currents create a stable equilibrium state, the electromagnetic horizontal force acts as a controller, with the stable equilibrium position as the reference position. This system consists of the electromagnetic controller and the load elements of the plant, as represented in Figure 5. This electromagnetic controller does not require a positioning sensor.

Nonetheless, this electromagnetic sensorless controller presents many limitations. The first one is the working range; it works only inside the range of 1 pitch (29.778 mm). That is because each combination of phase currents creates a sinusoidal distribution of the forces along the axis, with one stable equilibrium position in each pitch. Thus, the electromagnetic horizontal force takes the motor to the nearest stable equilibrium position, which may be in a maximum distance of ±14.889 mm. Another limitation of the electromagnetic controller is that it does not allow the tuning of the transient response. However, these two limitations can be overcome by introducing, as an input position (x_ref_), a discrete ramp that moves the motor in small steps until it arrives at the target position. This allows control of the movement from the initial position to the target position, working in the full range of the linear motor.

The most significant disadvantage that cannot be overcome in this open-loop system is the positioning accuracy. The constant parameters k and A of the motor law (Equation (1)) have been determined theoretically and experimentally (Table 2). Nevertheless, the values of these parameters are an approximation. They may vary from point to point and from pitch to pitch as the motor is not ideal. Similarly, the generated phase currents may also present deviations. Hence, the electromagnetic controller will take the motor to the stable equilibrium position; however, due to these inaccuracies, the equilibrium position may not be exactly coincident with the target position.

## 6. One-Dimensional Control Strategy and Hardware Implementation

In the previous section, a dynamic characterisation was performed, and it was stated that the electromagnetic force could be defined to behave as a sensorless controller. Nevertheless, as mentioned, the electromagnetic sensorless controller presents many limitations: a working range of one pitch, positioning errors and uncontrolled transient response. Therefore, in order to detect the movement errors, a positioning sensor should be implemented. The readouts of this positioning sensor can be used as feedback for a proportional–integral–derivative (PID) position controller that compares them to the reference position and defines the action necessary to correct the error. Moreover, it will allow the full travel range of the motor to be used. Finally, by tuning the PID controller, it is also possible to adjust the transient response.

**Figure 6:**
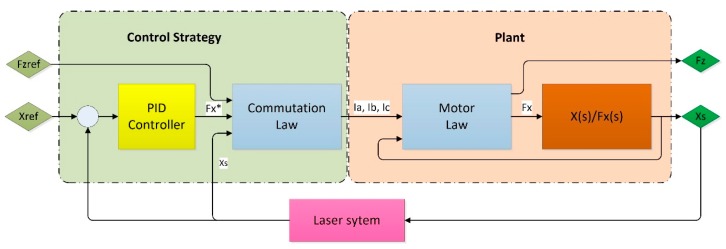
Position control scheme of the linear motor.

The resultant position control system has been represented in Figure 6. The reference position (x_ref_) is the input to the PID controller, whose output is the required horizontal force (F_x_*). Knowing the desired vertical force (F_zref_) and the required horizontal force (F_x_*), the commutation law calculates the required phase currents that are needed to generate those forces at the present position (x_s_). The resultant phase currents are generated by the control hardware and, according to the motor law, the electromagnetic forces F_x_ and F_z_ are produced. The horizontal force F_x_ displaces the motor to the desired position while F_z_ favours the levitation. The real position of the motor is read by a positioning sensor and fed back to the PID controller where it is compared to the reference position and corrected. The positioning sensor readouts are also used as inputs to the commutation law.

Once the 1D control strategy has been developed, it can be implemented in the control hardware and the positioning sensor that were presented in Section 4. The control hardware that has been chosen is a DMC Kit from Texas Instruments, and a Renishaw XL-80 laser system acts as positioning sensor. In this section, the implementation of the control system in the control hardware is presented and schematized.

The selected control hardware presents many advantages, such as being commercial, having a low cost and being suitable for this application. However, it is generic hardware for the control of rotary motors, where the main target is to control the rotation speed or the torque. Therefore, it has some limitations that need to be taken into account.

The first drawback is that the three phases must be wired, presenting a start connection. This constraint does not allow the introduction of an additional constraint of minimum power losses, as mentioned in Section 3.2, when the commutation law was defined.

In addition, the control hardware is not able to act on the phase currents directly as in [21,25], where hardware with current amplifiers was built for the control. Instead, it generates phase voltages by pulse wide modulation (PWM) [27]. The control program must calculate the duty cycles (DC) that are required to generate a desired phase voltage. Then, the transistor bridge generates the pulses of the PWM according to the DC, producing the corresponding voltages of each motor phase. The resulting phase currents flowing through the phases are constant, and their value depends on the phase voltages and the winding phase resistances that, in this case, are approximately 1 Ω for every phase. The DMC Kit from Texas Instruments includes high-resolution PWM (HRPWM) modules based on micro-edge positioner (MEP) technology which are able to extend the time resolution capabilities of the conventionally derived digital pulse [28]. The PWM working frequency must be higher than 10 kHz, according to the manufacturer. In order to get the best DC resolution, it was set to 14.64 kHz. The phase voltage resolution obtained when using the HRPWM modules at this frequency is 2.64 × 10^−5^ V.

Besides this, the control card is able to communicate in real-time with other peripherals, such as a computer, and transmit data through the serial communication interface (SCI).

**Figure 7:**
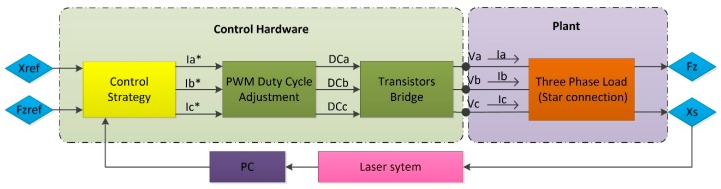
Implementation of the one-dimensional (1D) control strategy in the control hardware.

Figure 7 presents the control system implementation in the control hardware, having the laser system as positioning sensor. The control strategy reference inputs are the desired position (x_ref_) and the vertical force (F_zref_). The outputs are the required phase currents (I_a_*, I_b_* and I_c_*). As already mentioned, the control hardware does not act directly on the phase currents. Instead, it generates phase voltages by PWM. Hence, the PWM modules must generate the phase voltages that correspond to those phase currents. The required DCs must be calculated for the transistor bridge to generate these phase voltages. The voltage drop between the phase terminals and the neutral point of the motor creates the phase currents (I_a_, I_b_ and I_c_). Thus, by the motor law, two orthogonal forces (F_x_ and F_z_) are generated, and their magnitude depends on the relative position between the stator and the magnet array (x_s_). The motor position is measured by the laser system, and the readouts are extracted to the PC and directly sent to the control card through the SCI, together with the reference position command (x_ref_). The control strategy is performed at the sampling speed of the positioning sensor. In this case, the fastest sampling speed of the laser system is 0.05 s.

## 7. Experimental Results

This section presents and analyses the performance of the implementation of the developed electromagnetic sensorless controller and the 1D control system, which were previously described. The aim of the experiments is to confirm that the 1D control system fulfils the working conditions that the NanoPla design demands. For every experiment, the vertical force value defined as reference is 1 N, which defines the phase currents working range, that, in this case, is ±0.5 A.

### 7.1. Electromagnetic Sensorless Controller Results

Firstly, the electromagnetic sensorless controller that was presented in Section 5.2 was implemented and analysed. As expected, the electromagnetic controller is able to perform displacements inside a range of ±14.889 mm. The repeatability of the system when performing the same displacement of 5 mm inside the same pole 10 times is ±0.018 mm, and the average positioning error when reaching the position of 5 mm is 0.757 mm. The cause of this variation when performing the same displacement is that the electromagnetic controller displaces the motor to a certain position by creating a stable equilibrium state at this point. The stable equilibrium position is defined by the combinations of phase currents. Even though the command for these currents does not vary for the same target position, the real resultant phase currents may not be the same as they depend on other factors, such as the voltage generation noise and the winding resistor, which may vary with the temperature.

**Figure 8:**
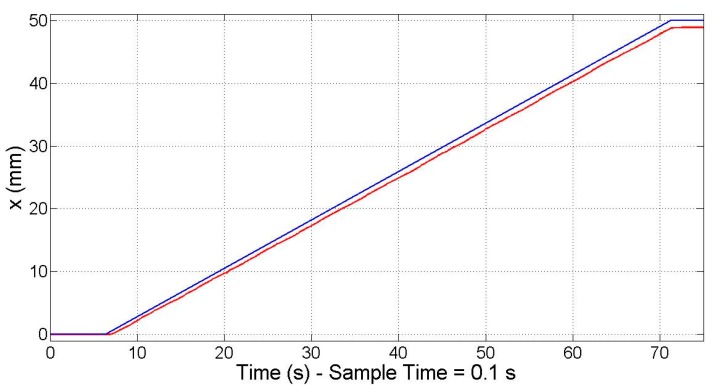
Electromagnetic sensorless controller: 50 mm travel range at constant speed.

By introducing a stepped ramp as the input for the reference position, it was confirmed that the electromagnetic sensorless controller is able to work in the full range of 50 mm (Figure 8) as stated in Section 5.2. Nevertheless, as expected, this sensorless controller is unable to correct the positioning error, which increases the farther it moves away from the zero position. At the end of the travel range, when the reference position (blue line) is 50 mm, the real position (red line) of the motor is 48.920 mm, which supposes a positioning error equal to 1.080 mm. This positioning error is not acceptable for the NanoPla operation, and thus another control strategy approach is necessary, such as the one proposed below.

### 7.2. One-Dimensional Control Strategy Results

The control system was implemented in the control hardware and its PID was experimentally tuned. As was done in the previous subsection for the electromagnetic sensorless controller, the repeatability of the system was measured by performing the same displacement of 5 mm 10 times. In this case, the system always reaches the target position; that is, its repeatability is equal to 0, and the average position error is 0 µm. Nevertheless, when the motor is at a stationary state, it slightly oscillates around the target position. This positioning noise has a root mean square (RMS) deviation of ±0.143 µm.

**Figure 9:**
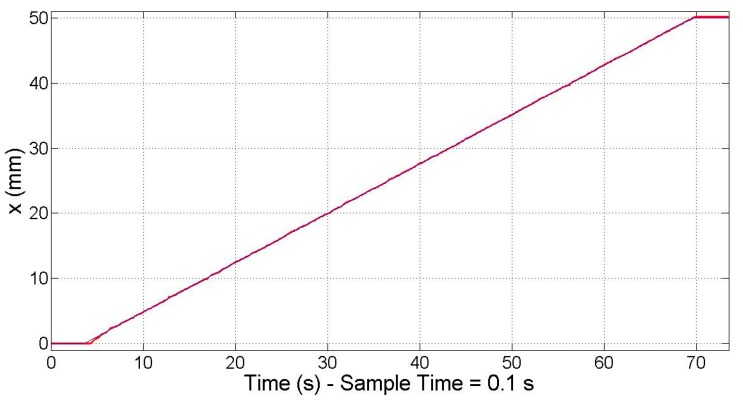
Closed-loop proportional–integral–derivative (PID) controller: 50 mm travel range at constant speed.

In order to confirm that the control system works along the travel range of 50 mm that is required in the NanoPla, the response to a 50 mm travel range input at a constant speed was recorded. As can be seen in Figure 9 the measured position (red line) follows the reference position (blue line) along the travel range, even doubling the sample time to 0.1 s.

**Figure 10:**
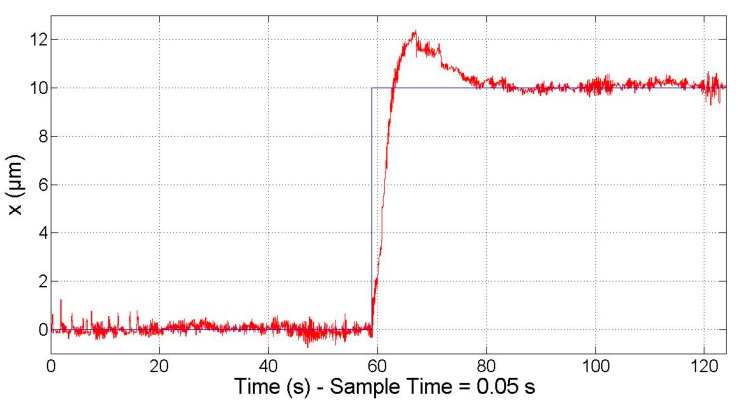
Closed-loop PID controller: 10 µm step response.

Moreover, it was verified that the motor is able to respond to the minimal required motion, that is, 10 µm, as stated in the Introduction. In Figure 10, the response (red line) to a 10 µm step (blue line) is represented.

**Figure 11:**
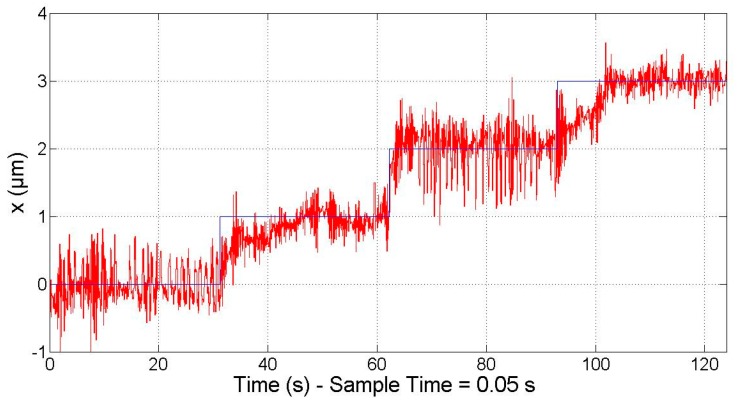
Closed-loop PID controller: 1 µm staircase response.

Besides this, the smallest step input that the motor can generate was also tested; that is, the minimum incremental motion. It must be noted that, in order to perform the motion, a change in the phase currents must occur. In turn, the variation of the phase currents is produced by a variation of the phase voltages that are controlled by the HRPWM module. As mentioned in the previous section, the minimum voltage variation that this module can perform is 2.62 × 10^−5^ V, which corresponds to a variation of approximately 2.62 × 10^−5^ A in the phase currents. Ideally, a change of this magnitude in the phase currents produces a displacement of approximately 600 nm. Nevertheless, the phase currents are also affected by the noise of the voltage source and the PWM signals. Therefore, the power stage is not able to work in the full range of the needed phase currents to perform a motion step in the submicrometre scale. However, the PID controller is fast enough to switch between two combinations of phase currents in order to reduce the positioning error, resulting in an improvement of the effective motion resolution. As shown in Figure 11, the system is able to respond to a staircase of 1 µm (reference position in blue and response in red). The magnitude of the positioning sensor measuring noise together with the resolution and noise of the voltage generation are the main contributors to the positioning noise of the control system. It must be taken into account that the laser system used for the experimental validation has a measuring noise in the range of 400 nm. In contrast, the laser system of the NanoPla has a measuring noise in the range of 20 nm. Therefore, better results are expected when the driving system is implemented in the NanoPla.

It should be noted that this results are for the motor under study, the design parameters of which are represented in Table 2. Experimental tests have shown that other motors having similar design parameters provide the same results. Nevertheless, for other values of k and A in the motor law (Equation (1)), the results could be different. The main aspect that should be taken into account when implementing this control system is the phase current’s working range and the minimum variation in the phase current that the voltage generation module is able to perform. The phase current’s working range is defined by the design parameters of the motor and the reference vertical force, while the minimum variation in the phase current is limited by the hardware.

In the control strategy, a reference value is also set for the generated vertical force (Figure 6). As mentioned, although the levitation of the moving platform of the NanoPla is performed by three air bearings, the design includes the use of the vertical force generated by the motors to favour the levitation. The inverted stators placed on the superior base of the NanoPla will attract the magnet arrays fixed to the moving platform. However, most of the load will be supported by the air bearings, and the linear motors will provide a levitation force of 1 N each. In the experimental setup, the vertical force generated by the motor was measured with a load cell. When the motor arrives to the target position, the vertical force is positive and constant, as required. However, when the motor moves from the initial position to the target position during the transient period, the vertical force varies slightly. It was observed that the transient response of the vertical force improves with the closed-loop control system compared to the open-loop system. During the transient response of the open-loop system, the value of F_z_ decreases by 18%. In contrast, in the closed-loop system, the value of F_z_ increases by 7%. According to the manufacturer of the air bearings, considering an air gap of 5 µm, they have a stiffness of 13 N/µm. Therefore, a change of 0.07 N in the load will compress the gap by 5 nm, which is acceptable for the application.

## 8. Conclusions

In this work, a control system for a Halbach linear motor in 1D has been designed, implemented and experimentally validated in commercial control hardware. The chosen hardware is a Digital Motor Control Kit from Texas Instruments. The usual application of this DMC Kit is the control of the rotation speed or the torque in rotatory motors. As a novelty, in this work it is used to control the position of a linear motor.

The developed control system will be implemented in the four Halbach linear motors that work as actuators in a 2D-nanopositioning stage (NanoPla). The NanoPla is currently in development at the University of Zaragoza. Halbach linear motors have been chosen as actuators because they allow movement along the moving axes and, also, in the orthogonal direction. Therefore, the 2D movement of the NanoPla is achieved in one plane. In addition, besides the propulsion force, Halbach linear motors generate a vertical force that favours the levitation of the moving part of the NanoPla. Developing the control system in a commercial hardware facilitates the future industrial applicability of the NanoPla.

Firstly, this work has proposed an open-loop control system that uses the electromagnetic horizontal force generated by the motor as a controller. Being a sensorless control system, it presents positioning errors that cannot be corrected. Consequently, a laser system has been chosen as a positioning sensor, and a closed-loop control system has been designed. Then, the developed control system has been implemented in the chosen hardware. The limitations of the commercial hardware have been overcome by optimizing the design.

Once the control system was designed and implemented, its performance was validated in the experimental setup. It has been verified that the system fulfils the working requirements of the NanoPla, which is a working range of 50 mm and a step response of 10 µm. It was also tested that the system is able to respond to steps of 1 µm. It must be noted that the laser system used in the experimental validation has a noise of 400 nm, while the laser system of the NanoPla has a noise of 20 nm. Therefore, the performance is expected to improve in the NanoPla. Additionally, the vertical force generated by the motor has also been measured, being constant at steady state and varying slightly (+7%) during the transient period; that is, when the motor is moving from the initial position to the target position. In the NanoPla, the main support of the levitation of the moving platform comprises three air bearings, while the motors favour the levitation with a force of 1 N each. Thus, a variation of 0.07 N does not affect the stability of the system.

The fact that the developed control strategy implemented in the chosen control hardware is able to operate according to the NanoPla design requirements, make unnecessary the use of more advance control devices. In future works, the control system that has been presented in this paper for one Halbach linear motor along one dimension will be implemented in the four NanoPla actuators to provide a two-dimensional travel range. One DMC Kit will be needed for each motor and the whole control strategy and experimental results will be obtained to assure the desired performance requirements of the NanoPla. At this point, different variants of the control strategy should be tested, in order to find the best performance. These variants could be for instance, operating at constant speed or applying a feedforward loop.

## Figures and Tables

**Table 1:** Description and theoretical values of the motor parameters.

Parameter	Description	Theoretical Value
N_m_	Number of spatial periods of the magnet array	2
η_0_	Winding density of the stator coil	832,400 turns/m^2^
μ_0_M_0_	Remanence of the permanent magnets	0.4 T
G	Effects of the motor geometry	2.62 × 10^−6^
k	Fundamental wave number	211.1285 rad/m
z_0_	Separation gap between stator-magnets array	400 µm
l	Spatial period of the array wavelength	29.76 mm

**Table 2:** Theoretical and experimental fitting parameters of the motor law.

Parameter	Theoretical Value	Experimental Value
A (N/A)	1.6	1.6067
k (rad/m)	211.1285	211.0001
l (mm)	29.760	29.778
